# Effects of Heat Stress on Gut Microbiome in Rats

**DOI:** 10.1007/s12088-021-00948-0

**Published:** 2021-05-29

**Authors:** Qian Qu, Hua Li, Lin Bai, Shiwei Zhang, Jiaqi Sun, Weijie Lv, Chunxin Ye, Cui Liu, Dayou Shi

**Affiliations:** 1 College of Veterinary Medicine, South China Agricultural University, Guangzhou, 510642 People’s Republic of China; 2 The Second Affiliated Hospital of Guangzhou Medical University, Guangzhou, 510260 People’s Republic of China

**Keywords:** Gut microbiome, Heat stress, Metabolism, Rats

## Abstract

Gut microbiome, as the largest and most important micro-ecosystem, plays a critical role in health. The purpose of this study was to evaluate whether heat stress modulates the composition and diversity of the gut microbiome in rats. The heat stress model was prepared in rats with the heating temperature maintained at 35–38°C. Cecum contents were collected after heat stress for 3 h and days 1, 3 and 7. Total DNA was extracted for 16 S rRNA sequencing and analysis of intestinal microbiome composition and diversity. The study showed that the composition of the intestinal microbiome of heat stress group was changed. And the heat stress modulated key phylotypes of gut microbiota at the level of phylum and genus. In particular, the genus of *Lactobacillus* and *Bacteroides* were significantly reduced, whereas the *Oscillospira* and *Clostridium* were increased by heat stress. Meanwhile, the rats under the heat stress encountered the change in carbohydrate metabolism, amino acid metabolism, and membrane transport to defense against stress. Taken together, the composition and structure of gut microbiome were affected by heat stress and some key phylotypes were also significantly altered. We conclude that the heat stress could impact multiple biological functions, via altering the gut microbiome.

## Introduction

Heat stress resulting from elevated ambient temperature is considered to be one of the most important environmental stresses exerting deleterious effects on homeostasis and severe systemic inflammatory response [1–3]. Heat-induced multiple negative effects on physiological, immune function, central nervous system, gut microbiota and reproductive functions [4–7]. The heat exposure to mice also causes changes in epithelial barrier dysfunction and cell structure, which is the key to colonize gut microbiota [8–10]. Heat stress can cause heatstroke, which is characterized by hyperthermia and central nervous system dysfunction [11]. Under stress, a reduction in intestinal villus height, an increase in crypt depth, and the change of intestinal villus can lead to the weakening of the intestinal absorption and the dysbiosis of the intestinal microflora [9, 12, 13]. Over the past few decades, the mechanism of heat stress is considered to be caused by the disorder of central nervous system, not by peripheral organs. And the understanding of intestinal function also focuses on the digestion and absorption of nutrients [14–17].

Microbiota inhabiting the mucosal surface of the animal skin, mouth, gastrointestinal tract and other organs is considered as the largest and most important micro-ecosystem. Gut microbiota residing in the digestive tracts of humans and other animals is the largest and complex community of microorganisms. Gut microbiome play diverse roles in animal and human physiology, including digestion of fiber, starch [18]; degradation of carbohydrate fermentation to produce lactic acid, short chain fatty acids, and other metabolites [19, 20]; synthesize certain vitamins (e.g., B1, B2, B12, VK, and folic acid) [21]; modulate immune system and functions [22, 23]; influence intestinal epithelial permeability [24, 25]; and prevent infections from other pathogens [26]. In addition, emerging evidence suggests that the gut microbiome also regulates brain development and behavior [27–29]. Studies have reported the change in the response of germ-free rats to stress, an abnormality in the hypothalamic pituitary adrenal axis adjustment, and the decrease in the pain caused by inflammation [30, 31]. This study mainly discusses the changes of heat stress on gut microbiome in rats and then provides a new possible mechanism of heat stress leading to inflammatory response and nervous system disorders.

## Material and Methods

### Animals

All experimental procedures in this study were approved by the Animal Ethics Committee of the South China Agricultural University (Guangzhou, China). The care and use of the animals were carried out under the Guidelines for Animal Experiments of the South China Agricultural University, and all efforts were made to minimize the number of animals, suffering, and to maximize their well-being (permit number: 2014321, Guangzhou, China). All procedures involving animals throughout the experiments were conducted in strict accordance with the Chinese legislation on the use and care of laboratory animals.

Seven-week-old male-specific pathogen-free (SPF) Wistar rats (180 ± 10 g, purchased from the Center of Experimental Animals of Southern Medical University, approval number: 44002100005777) were housed under standard conditions (22 ± 0.5 °C, 50 ± 5 % humidity, and a 12 h light/12 h dark cycle) and maintained with free access to a standard laboratory pellet diet and water.

### Experimental Design In Vivo

Forty adult Wistar male rats were randomly divided into 2 groups with 20 rats in each group: heat stress group (HS) for which the heater temperature is maintained at 35–38 °C and humidity values at 50 to 60%, and the control group is maintained at a temperature of 24–26 °C with free access of drinking water and food for 7 days. The rectal temperature was recorded and the contents in cecum from 5 rats were collected after euthanasia at 3 h and day 1, 3 and 7. And the blood was drawn from the abdominal aorta, and the resulting serum was used to measure the circulating level of white blood cell (WBC) and glucose (GLU) after clotting at day 7. All samples were stored in − 80 °C freezer until analysis. In this study, all rats were euthanized by 30 mg/kg pentobarbital sodium and cervical vertebra dislocation.

### Bioinformatics and Statistical Analyses

Each rat cecum contents were used to extract total DNA for 16 S rRNA sequencing and analysis of cecum intestinal microbiome. Then Illumina Miseq reads were analysed using FLASH software. Briefly, paired-read pairs were assembled into contigs that contained the V3–V4 Tags of 16 S rRNA [32]. All Tags need a stringent quality control processing. Any Tags with ambiguous base and shorter than 200 bp were culled. Identical or duplicate sequences were merged. Chimera sequences were checked and removed using usearch61. According to the similarity of sequences, Tags were clustered to (OTUs) [33]. OTUs were calculated at a distance of 0.03, using the UCLUST [34]. Alpha diversity includes Chao1 and Shannon index and rarefaction curves. The beta diversity analysis of PCoA based on Bray-Curtis was calculated by the weighted and unweighted Unifrac [35]. R was used to visualize the abundance of the bacterial taxonomic composition. Community function prediction is based on the KEGG database. The bar graph of the genus was produced with GraphPad Prism 5 software. The significance of variance was analysed by one-way ANOVA. And *represents *P* < 0.05 and **represents *P* < 0.01.

## Results

### Heat Stress on Rectal Temperature, White Blood Cell and Glucose

The rectal temperature (RT) was recorded in rats at 3 h and day 1, 3 and 7 (Table 1). There were obvious differences in the RT of rats during the study between the HS group and control group (*P* < 0.01). The circulating level of WBC and GLU was also measured on day 7 (Table 2). The level of WBC and GLU in the HS group weas markedly increased by heat stress on day 7. These data suggested that the model of heat stress was successfully established in this study.

**Table 1:** The effect of heat stress on rectal temperature

Index	Group	3 h	1 day	3 days	7 days
RT (°C)	HS	39.65 ± 0.06**	38.52 ± 0.04**	39.54 ± 0.14**	39.00 ± 0.24**
	Control	37.63 ± 0.29	37.00 ± 0.09	37.67 ± 0.12	37.78 ± 0.07

*Represents *p* < 0.05 and **represents *p* < 0.01 compared to the Con group

**Table 2:** The effect of heat stress on white blood cell and glucose

Time point	Group	WBC (×10^9^/L)	GLU (mmol/L)
7 days	HS	6.52 ± 0.22**	7.10 ± 0.70**
	Control	3.00 ± 0.48	2.98 ± 0.56

*Represents *p* < 0.05 and **represents *p* < 0.01 compared to the control group

### The Effects of Heat Stress on Alpha Diversity Index

The results on Alpha diversity index of the gut microbiota in rats showed difference between the two groups (Table 3). After heat stress for 3 and 7 days, the Chao 1 index of HS group was significantly decreased than that of the control group. The phylogenetic diversity (PD) whole tree index of the HS group was significantly lower than that of the control group on day 7 and the Shannon index of HS group was significantly lower than that of the control group on 3 h.

**Table 3:** Alpha diversity indexes of the gut microbiota in heat stress rats

Time point	Group	Chao1	PD_whole_tree	Shannon
3 h	HS	4316.25 ± 43.14	103.96 ± 0.49	7.27 ± 0.15*
	Control	5973.33 ± 264.48	131.50 ± 3.31	8.13 ± 0.11
1 day	HS	7984.30 ± 263.59	148.32 ± 5.11	8.07 ± 0.04
	Control	7003.53 ± 244.73	148.32 ± 5.08	7.67 ± 0.06
3 days	HS	6343.86 ± 229.98*	146.34 ± 2.49	8.04 ± 0.02
	Control	8101.88 ± 335.32	163.49 ± 2.50	8.41 ± 0.06
7 days	HS	4604.57 ± 215.91*	103.60 ± 3.22*	7.67 ± 0.07
	Control	6197.48 ± 192.84	134.86 ± 2.02	7.88 ± 0.05

*Represents *p* < 0.05 and **represents *p* < 0.01 compared to the control group

### The Effect of Heat Stress on the Composition of Gut Microbiome

**Fig. 1:**
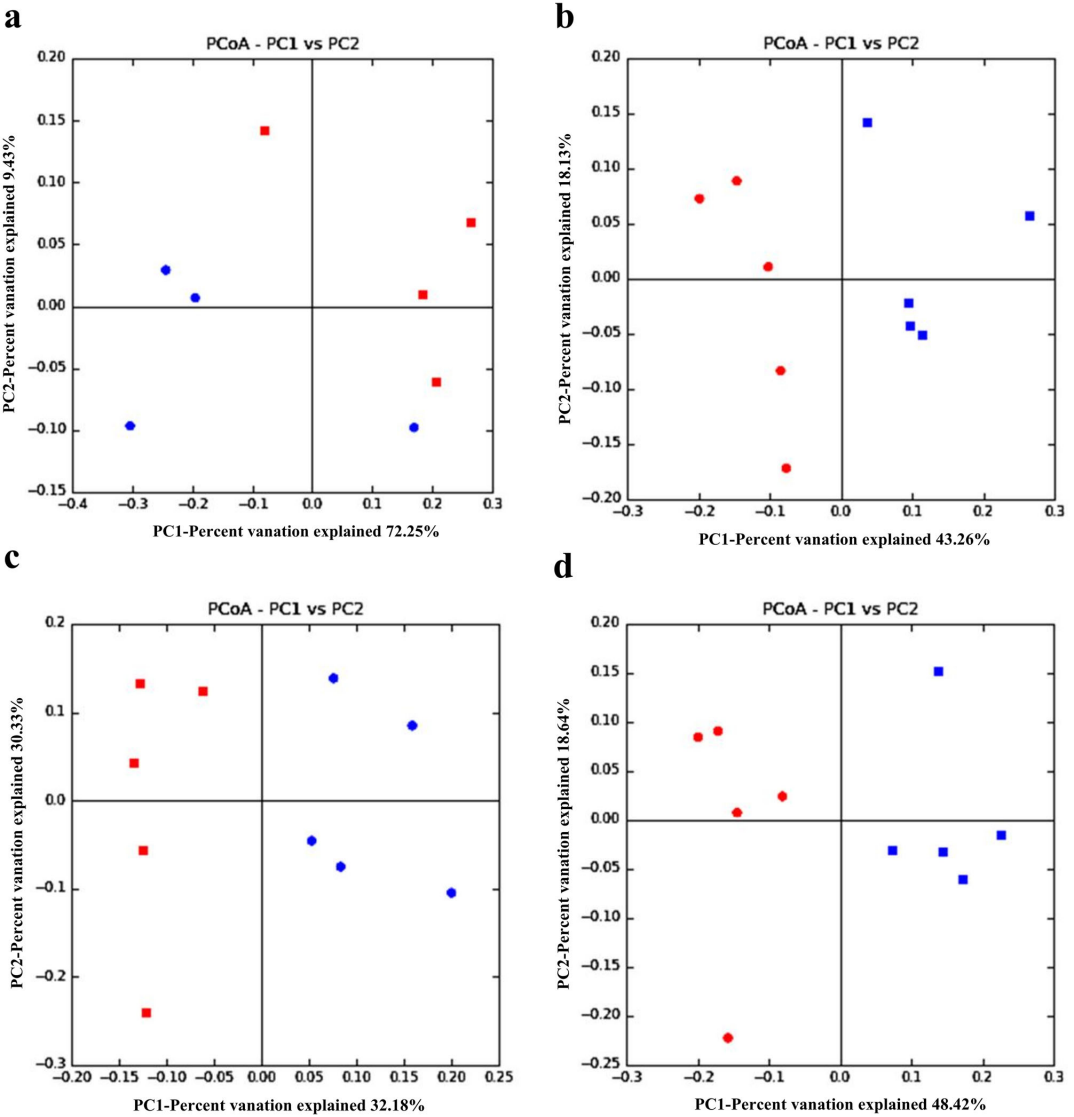
The composition changes of gut microbiota based on PCoA with weight Unifrac. **a** The PCoA with weight in 3 h; **b** the PCoA with weight at day 1; **c** the PCoA with weight at day 3; **d** the PCoA with weight at day 7. PC1and PC2 are the two principal coordinate components. The red plot is the HS group and the blue is the control group

**Fig. 2:**
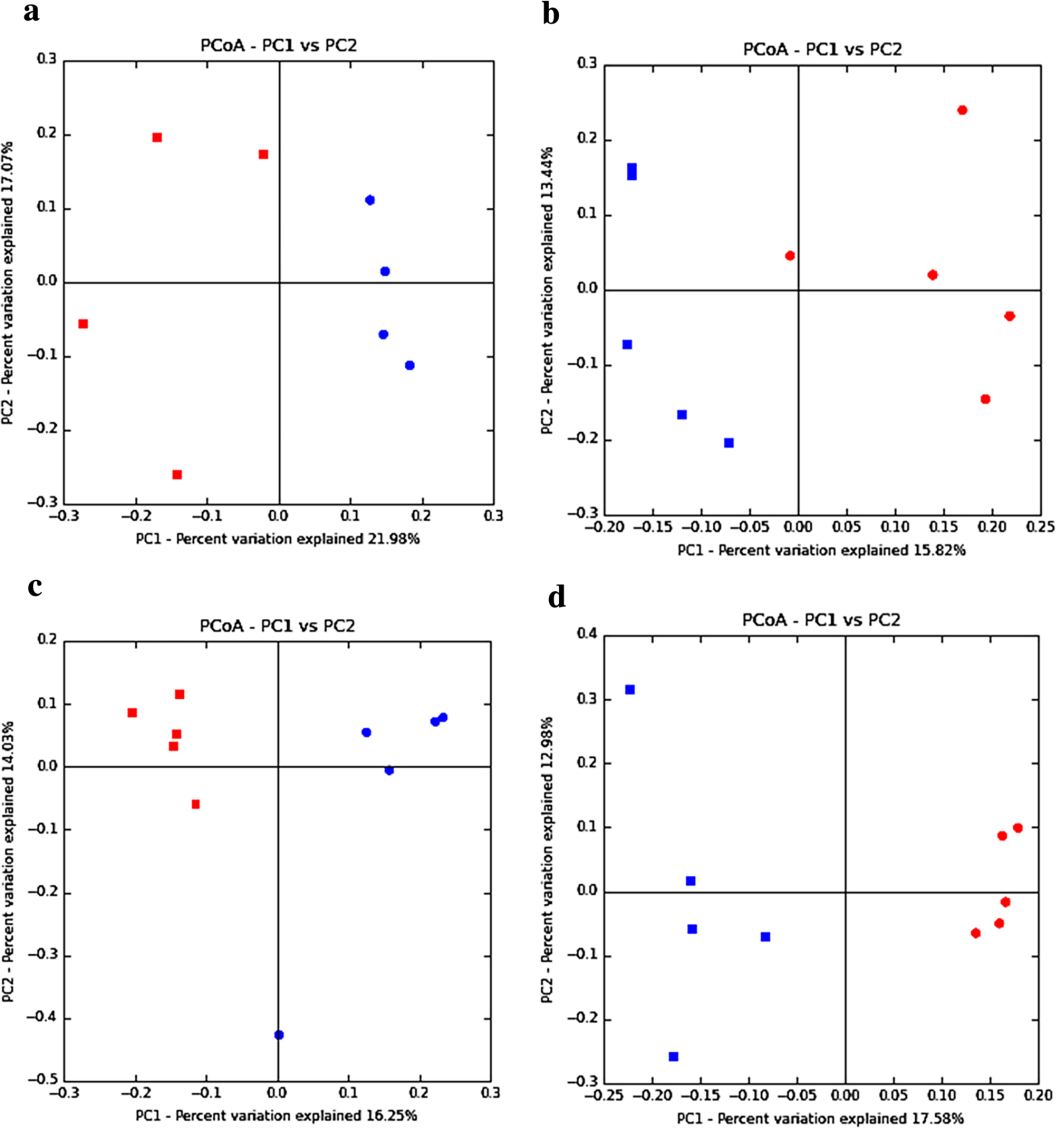
The composition changes of gut microbiota based on PCoA with unweight Unifrac. **a** The PCoA with unweight in 3 h; **b** the PCoA with unweight at day 1; **c** the PCoA with un weight at day 3; **d** the PCoA with unweight at day 7. PC1and PC2 are the two principal coordinate components. The red plot is the HS group and the blue is the control group

The principal coordinate analysis (PCoA) based on the Unifrac distances weighted (Fig. 1) and unweighted (Fig. 2) values was performed to determine the effects of heat stress on gut microbiome composition. In 3 h and on days 1, 3 and 7, the plot of PCoA were clustered together in the HS group and control group respectively, whereas the scatter points between the HS group and control group showed obvious differences, suggesting that the composition of the gut microbiome changes significantly in each stage by heat stress. These data suggest that the composition of gut microbiota in caecum is markedly changed by heat stress.

### The Community Contributed at the Phylum Level by Heat Stress

**Fig. 3:**
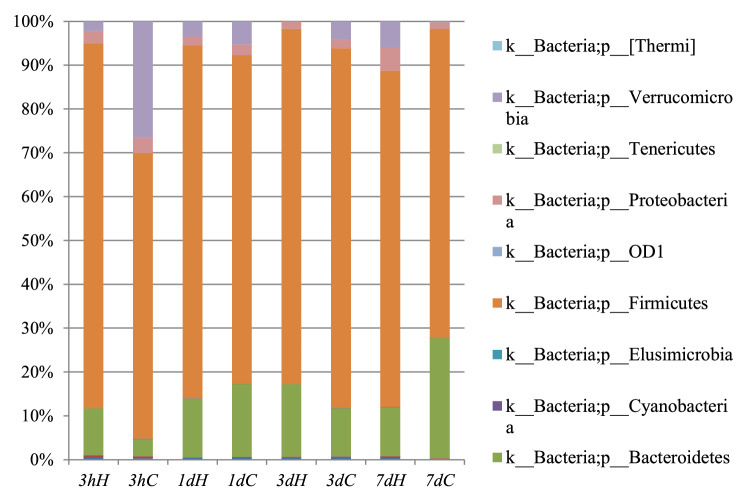
The relative abundance of gut microbiome at the phylum level by heat stress in rats. All phyla were defined as 1, and the proportion of each phylum is defined as percent relative abundance. H represents HS group and C represents control group

In this study, we used PyNAST software and Greengenes database to compare the known sequences and to compare the relative abundance at the phylum level in all the groups. At the phylum level, the relative abundance and community composition were significantly shifted by heat stress, whereas the dominant bacteria in caeca were *Firmicutes* and *Bacteroidetes* in all the groups (Fig. 3). The main phyla of *Firmicutes*, *Bacteroidetes* and *Verrucomicrobia* showed an obvious difference between the two groups. The relative abundance of *Firmicutes* was increased by heat stress except on day 3. In 3 h and on day 3, the phylum of *Bacteroidetes* was significantly increased than that in the control group, but decreased on days 1 and 7. The relative abundance of *Verrucomicrobia* was reduced in the HS group compared to the control group, but increased on day 7. These changed have no time-dependent and may be related to the dynamically changing gut microbiota.

### The Changes in Key Phylotypes at the Genus Levels by Heat Stress

**Fig. 4:**
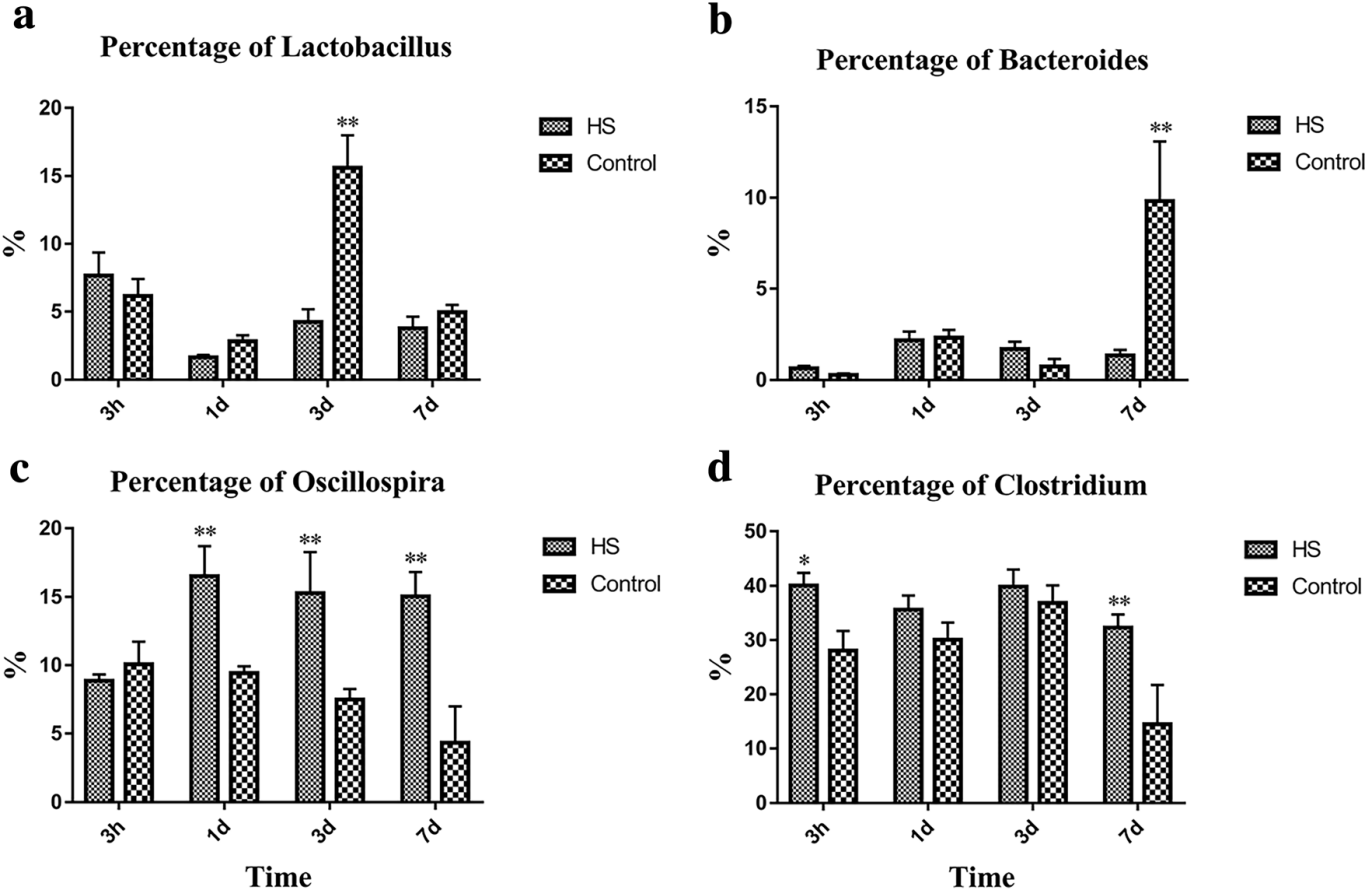
The relative abundance of gut microbiome at the genus level by heat stress. **a** The relative abundance of *Lactobacillus*; **b** the relative abundance of *Bacteroides*; **c** the relative abundance of *Oscillospira*; **d** the relative abundance of *Clostridum*. All genera were defined as 1, and the proportion of each genus is defined as percent relative abundance

Taxon-based analysis at the genus level showed that the relative abundance of *Lactobacillus*, *Bacteroides*, *Oscillospira* and *Clostridium* were significantly changed by heat stress (Fig. 4). The relative abundance of *Lactobacillus* was significantly reduced in the HS group compared to the control group on day 3 (*P* < 0.01), whereas the relative abundance of *Bacteroides* was reduced on day 7 (*P* < 0.01). For *Oscillospira*, the relative abundance was increased by heat stress on day 1, 3 and 7 and the *Clostridium* was also increased in 3 h (*P* < 0.05) and on day 7(*P* < 0.01).

### KEGG Community Function Prediction Annotation

**Fig. 5:**
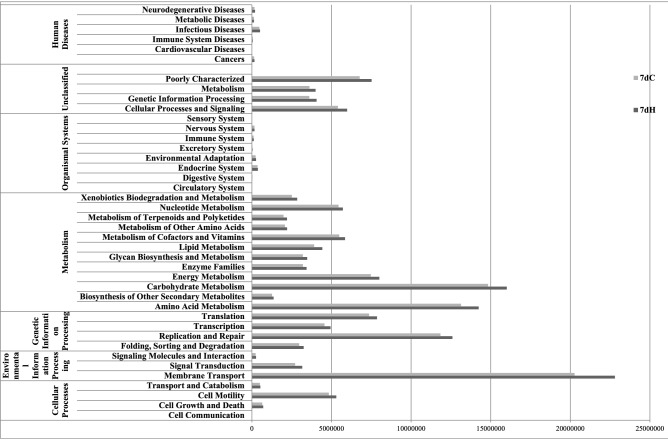
The KEGG community function prediction annotation by heat stress. 7dH is the HS group and 7dC is the control group at day 7

A total of 6909 functional genes were identified and have significant paired genes in the Kyoto, Encyclopedia of Genes and Genomes (KEGG) database. These pathways were divided into five main categories, including metabolic, genetic information processing, environmental information processing, cellular processes, and organismal systems (Fig. 5). Among the 5 functional categories, 35 major KEGG pathways were selected. The changes of biological pathways in the HS group mainly concentrated on the four types of pathways: metabolism, genetic information processing, environmental information processing and cellular processes. The HS group was more active in the carbohydrate metabolism, amino acid metabolism, and membrane transport gene expression compared with the control group, indicated that multiple biological functions of the body reaction path were shifted to be more active by heat stress and these changes may be related to the gut microbiome.

## Discussion

Heat stress can lead to the inflammation of the organ, including liver, hypothalamus, and intestinal bleeding, while the mechanism of heat stress based on the nervous system cannot be explained reasonably [5, 14, 36]. Recent Studies demonstrated that heat stress can worsen gut disorders and change the composition of gut microbiota, which may be one of the mechanisms of heat stress [37–39]. The results of this study suggested that the composition and structure of gut microbiota were affected by heat stress and the changes of biological pathways by heat stress may be related to the gut microbiota.

The previous study found that when the ambient temperature exceeds 28 °C, it can lead to the slight rupture and regional edema of the small intestinal (duodenum, jejunum, ileum) villus [10]. In this study, the temperature in our model of heat stress was in agreement with previous studies [1, 40]. And increased the rectal temperature, white blood cell and glucose were observed by heat stress in this study, which is in agreement with other studies of different animals [12, 41]. Taken together, the model of heat stress was established.

This study was conducted to explore the relationship between the composition of the gut microbiome and heat stress. Heat stress has adverse effects on growth performance, immunity and overall health, but its mechanism remains largely unclear, which may be related to gut microbiota [42, 43]. In this study, our results showed that the alpha diversity, including Chao1, PD whole tree and Shannon index of gut microbiota in cecum was significantly changed by heat stress. However, there was no significantly change on the alpha diversity index of day 1, which may be because the gut microbiota is a slow process, and it is consistent with the results of previous studies [7]. In this study, the Chao1 and PD whole tree first increased and then decreased upon heat stress, which may be due to the suddenness of stress stimulation. With the extension of stress time, the adaptation to the stress and gut microbiota of heat stress rats tended to be stable. The Chao1, PD whole tree and Shannon index based on operational taxonomic units (OTU), which is used to estimate the richness and diversity of gut microbiota and predict the number of species, were obvious decreased in HS group compared with the control group, indicating that heat stress can reduce the richness and diversity of gut microbiota. These results are consistent with previous studies that heat stress can change the richness and diversity of gut microbiome [44].

Our results based on PCoA showed that heat stress could modulate the composition of gut microbiome in caecum. Meanwhile, the obvious differences in the relative abundance of bacterial at the phylum and genus level between the HS group and Con group were obtained. Heat stress was previously shown to regulate the diversity of gut microbiota and inhibit the dominant *Lactobacillus* [45, 46]. And disruptive social, which is another stress, was also shown to reduce the relative abundance of the genus and increase the relative abundance of *Clostridium* [47].

In the current research, the phyla of *Firmicutes* and *Bacteroidetes* were the dominant bacteria and had a significant difference in the caecum of rats by heat stress, which was in agreement with another report, but in the fecal of laying hens [39]. The relative abundance of *Bacteroidetes*, which contains many beneficial genera, such as *Bacteroidetes*, was significantly decreased, whereas the *Verrumicrobia*, including *Akkermansia*, which can reduce body weight, was increased in the heat stress group, indicating that heat stress can affect the body by gut microbiota. At the genus level, the relative abundance of *Lactobacillus* and *Bacteroides*, which were putative beneficial genera, was significantlty reduced, whereas the *Oscillospira* and *Clostridium* were increased by heat stress. The finding of *Bacteroides* is consistent with our previous study on chicken, whereas the result of Oscillospira is contrary [7]. Results of KEGG community function prediction annotation demonstrated that the carbohydrate metabolism, amino acid metabolism, and membrane transport gene expression was more active with heat stress. *Lactobacillus* produce acetylcholine and γ-amino butyric acid, *Bacteroidetes* produce γ-amino butyric acid and are important in glucose homeostasis of hosts; and *Oscillospira* are the butyrate producer, which may be the key for the gut microbiome to affect host metabolism and biological functions [48, 49]. Gut microbiota and their secretions affect the nervous system, and regulate the intestinal motility and sensory afferent signal to the brain [50]. Central nervous system and neuroendocrine activity, in particular stress response, may in turn affect the composition of gut microbiota by changing the abundance of bacterial species and bacterial virulence factors [51]. Diseases can affect the composition of gut microbiome, and then in turn leading to deterioration of the body, which may be a process of circulatory action.

## Conclusions

In conclusion, our results showed that the structure of gut microbiome in rats was altered by heat stress. Particularly, the key genera of *Lactobacillus*, *Bacteroides*, *Oscillospira* and *Clostridium*, which can produce metabolites, were changed by heat stress. Consequently, based on KEGG, multiple biological functions of the body reaction path were regulated. Taken together, one of the mechanisms of heat stress may be the altered gut microbiota, which may lead to the change of metabolism, and then affect the biological function and nervous system. Future research will be needed to understand the role of metabolites produced by gut microbiome in the brain gut axis.

## Data Availability

All data generated or analysed during this study are included in this published article.

## Abbreviations

HS: Heat stress
Con: Control
RT: Rectal temperature
WBC: White blood cell
GLU: Glucose
PD: Phylogenetic diversity
PCoA: Principal coordinate analysis
OUT: Operational taxonomic units
KEGG: Kyoto, Encyclopedia of Genes and Genomes
